# Being Ready to Treat Ebola Virus Disease Patients

**DOI:** 10.4269/ajtmh.14-0746

**Published:** 2015-02-04

**Authors:** David M. Brett-Major, Shevin T. Jacob, Frederique A. Jacquerioz, George F. Risi, William A. Fischer, Yasuyuki Kato, Catherine F. Houlihan, Ian Crozier, Henry Kyobe Bosa, James V. Lawler, Takuya Adachi, Sara K. Hurley, Louise E. Berry, John C. Carlson, Thomas. C. Button, Susan L. McLellan, Barbara J. Shea, Gary G. Kuniyoshi, Mauricio Ferri, Srinivas G. Murthy, Nicola Petrosillo, Francois Lamontagne, David T. Porembka, John S. Schieffelin, Lewis Rubinson, Tim O'Dempsey, Suzanne M. Donovan, Daniel G. Bausch, Robert A. Fowler, Thomas E. Fletcher

**Affiliations:** Naval Medical Research Center, Silver Spring, Maryland; Uniformed Services University, Bethesda, Maryland; University of Washington, Seattle, Washington; Tulane University Health Sciences Center, New Orleans, Louisiana; Infectious Disease Specialists, PC, Missoula, Montana; Division of Pulmonary and Critical Care Medicine, The University of North Carolina at Chapel Hill, North Carolina; Division of Preparedness and Emerging Infections, Disease Control and Prevention Center, National Center for Global Health and Medicine, Tokyo, Japan; Clinical Research Department, London School of Hygiene and Tropical Medicine, London, United Kingdom; Infectious Diseases Institute, College of Health Sciences, Makerere University, Kampala, Uganda; Uganda Peoples Defence Forces, Kampala, Uganda; Naval Medical Research Center- Frederick, Fort Detrick, Maryland; Austere Environment Consortium for Enhanced Sepsis Outcomes (ACESO), Fort Detrick, Maryland; Toshima Hospital, Tokyo, Japan; Providence St. Patrick Hospital, Missoula, Montana; Department of Infectious Diseases, Nottingham University Hospitals, National Health Service Trust, Nottingham, United Kingdom; Truman Medical Centers, Kansas City, Missouri; Toronto, Ontario, Canada; The Queen's Medical Center, Honolulu, Hawaii; Department of Community Health Sciences, University of Calgary, Calgary, Alberta, Canada; University of British Columbia, Vancouver, British Columbia, Canada; National Institute for Infectious Diseases, Lazzaro Spallanzani, Rome, Italy; Centre de Recherche, Clinique Centre Hospitalier Universitaire de Sherbrooke, Sherbrooke, Quebec, Canada; Department of Medicine, Sanford School of Medicine, University of South Dakota, Sioux Falls, South Dakota; Avera McKenna Medical Center, Sioux Falls, South Dakota; Critical Care Resuscitation Unit, R. Adams Cowley Shock Trauma Center, University of Maryland School of Medicine, Baltimore, Maryland; Liverpool School of Tropical Medicine, Liverpool, United Kingdom; Division Infectious Diseases, Olive View UCLA Medical Center, David Geffen School of Medicine at UCLA, Los Angeles, California; U.S. Naval Medical Research Unit No. 6 (NAMRU-6), Lima, Peru; University of Toronto, Toronto, Ontario, Canada

## Abstract

As the outbreak of Ebola virus disease (EVD) in West Africa continues, clinical preparedness is needed in countries at risk for EVD (e.g., United States) and more fully equipped and supported clinical teams in those countries with epidemic spread of EVD in Africa. Clinical staff must approach the patient with a very deliberate focus on providing effective care while assuring personal safety. To do this, both individual health care providers and health systems must improve EVD care. Although formal guidance toward these goals exists from the World Health Organization, Medecin Sans Frontières, the Centers for Disease Control and Prevention, and other groups, some of the most critical lessons come from personal experience. In this narrative, clinicians deployed by the World Health Organization into a wide range of clinical settings in West Africa distill key, practical considerations for working safely and effectively with patients with EVD.

An unprecedented number of health care professionals from a variety of clinical settings, in a wide range of countries are thinking about, preparing for and caring for Ebola virus disease (EVD) patients. Guidance documents on infection prevention and control (IPC) practice and clinical care have been produced by organizations with EVD experience.^1^–^3^ The World Health Organization (WHO) produces guidance for implementation across a wide range of resource settings. Medecin Sans Frontières produces guidance for medical team activities across the outbreak. The Centers for Disease Control and Prevention (CDC) focus on measures which can be taken by the United States health system and extrapolated by others involved in preparedness and response. There are no short cuts to clinical preparedness for EVD. These documents and their revisions should be reviewed carefully.

As important as guidance documents are, many lessons must be learned from specific hands-on experience. The WHO has mobilized clinical consultants in support of EVD response in each of the affected countries in West Africa. This short list of key points attempts to consolidate practical lessons learned that do not always percolate into technical documents. Having landed in unconstrained, resource-limited settings at the start of local EVD clinical operations in an outbreak, and more established EVD care centers, we hope that others might adopt some of these lessons and avoid some of the risks inherent to the steep learning curve associated with delivering EVD care. The points are geared toward the daily care of patients as opposed to the critical mechanics of establishing a care center and developing its procedures. They are focused on the outbreak setting and also have relevance to the referral hospital setting.

## Be guided by the science

**Figure 1. F1:**
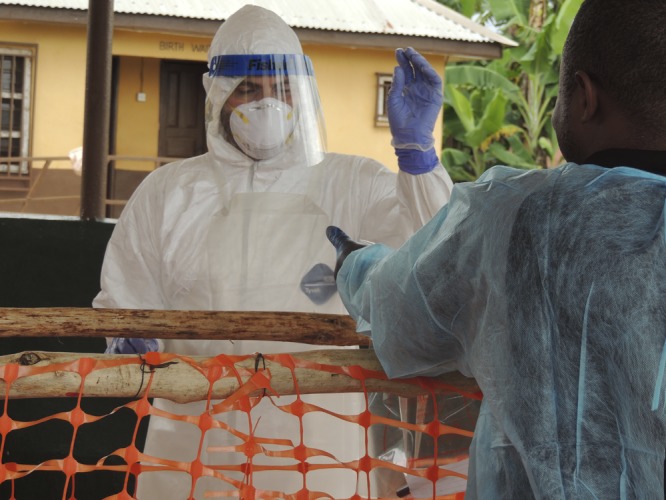
One of the authors delivers a suspect EVD case patient's diagnostic blood sample to a local healthcare colleague waiting in the low-risk area.

EVD patient care must be deliberate and vigilant. Anxiety around EVD reflected in media reports or shown by communities directly, and rapidly evolving events on the ground, sometimes blur facts. The science behind basic aspects of how clinicians can safely approach the patient in these settings should be respected. It is based on decades of laboratory research and field observation. Although much remains to be discovered, Ebola virus is spread only during the symptomatic phase of illness, especially in the setting of diarrhea, vomiting, or bleeding. Although the longest incubation period is 3 weeks, most cases present in < 2 weeks.^4^ Safe and effective care is possible and has been achieved repeatedly, in resource fortunate and resource poor settings. To do so, steps must be taken to ensure appropriate training and safe working conditions.^5^ These steps must be shaped by science and experience and not undermined by anxiety. Ebola virus, like all micro-organisms, possesses and follows defined physical and biological principles. Understanding these principles helps to eliminate a sense of mystery, reduces stress, and keeps responders' focus on the work (Figure 1).[Fig F1]

## Think aggressively; act safely

Safe and effective care for EVD patients has been achieved in both resource-poor and well-resourced settings. A targeted strategy of aggressive volume repletion and electrolyte management, vigilance for patient safety in the isolation environment, attention to hypoperfusion-related complications and co-infection (particularly malaria among those from endemic areas), and general supportive management for hospitalized patients improves survival.^5^ Both oral rehydration solution (ORS) and parenteral fluid and electrolyte resuscitation can be given aggressively and safely while following standard, contact and droplet infection prevention and control precautions. Peripheral and central venous access, dialysis, and mechanical ventilation have been performed safely in the right settings.

All procedures in the high-risk area, just as in patient care everywhere, must follow a careful risk-benefit assessment. “First do no harm” applies to the patient, the staff, and the community. Taking a few minutes before conducting any intervention to ensure the procedure will benefit the patient, and is adequately prepared with the necessary materials, support, and environment, may dramatically enhance staff safety. Team approaches enhance the preparation and execution of procedures. Procedures should be appropriate to the mix of need and resources at hand. They should be practiced. For each procedure, refine movements so that they are deliberate and carefully consider the placement of supporting staff.

## Ensure that the working environment is safe for both you and your patients

EVD care must occur in a work environment that draws on non-clinician expertise. Clinicians typically do not manage environmental aspects of health facilities. In EVD care, they have a critical stake in it. For instance, the health unit where EVD patients will be treated requires a thoughtful layout taking into consideration staff and patient flow through low- and high-risk areas, sufficient numbers of staff (clinician and non-clinician), and robust water and sanitation, hygiene, and waste management support.

## Be confident but careful in the use of personal protective equipment

Personal protective equipment (PPE) protection requires careful and comprehensive training, repeated practice, and competency assessment. This must be in the context of on-site IPC and clinical procedures that are safe, sensible, functional, and reproducible. Training and practice must occur before and during work with suspect or confirmed EVD patients. Mentoring by more experienced clinicians is critical. Systems should include a designated controller of the doffing and decontamination area, and constant co-supervision of each other using a buddy system to reduce errors and the risk of infections.

## Anyone can and should call a safety stop

Everyone associated with running an EVD care center, in and out of high-risk areas, is responsible for contributing to safe and effective patient care. Regardless of job, rank, and culture, anyone can and should flag concerns for staff and patient safety. Sometimes having a single, universal word that anyone can say to freeze activity is helpful. Usually we use the word “stop.” It is not commonly used for other reasons. When a concern is present, “stop” reminds people to cease all movement and activity until the concern is voiced and addressed through a risk-benefit assessment. This approach also prevents multiple people trying to provide instructions at once—a common occurrence, which can increase a person's risk in doffing areas where multiple people are observing the removal of PPE.

## Protect and call “stop” on yourself

Proper IPC practice requires practice, patience, monitoring, assessment, and intervention. Donning and doffing of PPE, safe sharps use and disposal and patient movement procedures must be carefully rehearsed and not rushed. Almost inevitably, despite the best of preparations, a process occasionally will go wrong when in an isolation area. Visors, glasses, or goggles fog, face masks become saturated and collapse toward the nose or mouth, suits and gloves tear, light fades, power outages occur, a patient becomes agitated, fatigue or heat stress intrudes. When this happens to us, we stop, stand upright, place hands in a neutral position folded in front and take a few breaths. We then decide whether there really is a problem. If there is, we decide whether it inhibits completing the task at hand, whether we should finish that task or redesign it, or immediately exit the high risk area and safely doff PPE. Regularly ask yourself “is it safe for me to do this now?” When in doubt, exit expeditiously with your buddy system partner. Take fluids—many of us have been slow to take fluids aggressively enough—reassess the situation and either decide to get dressed again for short re-entry to complete a task or turn it over to someone else.

The PPE and its use is only part of good IPC practice. The PPE brings specific challenges. The removal of contaminated PPE presents risk and requires a structured process and attention to detail including real-time guidance and monitoring. The process of doffing will take many minutes and must not be rushed. This must be factored into both planning and the exit decision.

Complications with PPE are not the only set of challenges that may require a clinician to take stock of the situation acutely. An agitated patient may interrupt a needle procedure. A pause can allow some tasks to be redesigned such as changing to alternate routes of medication administration. Sometimes, critical interventions such as peripheral intravenous (IV) placement must be deferred until a subsequent entry into the high-risk area.

Patients and staff are far better served with more frequent entries into the high-risk area over time than single long entries that increase fatigue and the possibility of risky behavior. Time scheduled in PPE may need adjustment to fit the individual, climatic conditions, and the tasks. Remember to alert teammates when exiting. There is no shame in an unexpected exit.

## Time in personal protective equipment should be time with the patient

Patients require bedside clinician care. The most important aspect of clinical care is close interaction between the healthcare provider and the patient. In the field, this can be compromised by marked resource-need mismatches. Furthermore, even the most acclimatized professional with the best working conditions gets fatigued and potentially distracted in PPE while observing comprehensive IPC practice. Solid preparation and planning of activities in the high-risk area preserves time with the patient, increases general efficiency, and increases safety. For example, procedures requiring the use of sharps such as adding electrolytes to crystalloid solutions can be done before entering the high-risk area, preserving time with the patient and limiting use of sharps in a high-risk environment.

## Treat the patient, not the idea of the patient

Clinicians carry many preconceived notions about what a viral *hemorrhagic* fever patient looks like. In fact, hemorrhage is *not* a prominent sign or symptom in most patients presenting with EVD. Respect the clinical syndrome observed in the individual patient. Like any severely ill patient, an EVD patient requires objective and longitudinal evaluation and intervention. These patients can have waxing and waning clinical courses or precipitous deteriorations. All of us have been humbled by how quickly some EVD patients progress from being moderately stable to severely ill. Young patients can appear compensated longer before rapid declines. In part, this may be a result of barriers in achieving an optimal clinical examination in PPE and a lack of clinical laboratory testing in field settings. Nonetheless, patients in referral intensive care unit (ICU) settings with severe multiple organ dysfunction and requiring ventilator and dialysis support have recovered.

Although the dominant clinical challenge in most EVD patients is volume and electrolyte resuscitation, common non-infectious co-morbidities such as diabetes, hypertension, and heart disease may complicate disease course, particularly in older patients. In addition to malaria, other endemic health risks are present in West Africa including helminthic infections, amoebiasis, acute thiamine deficiency, and sickle cell disease. Patients and families remain the best sources of information. Many barriers exist to obtaining it. They include challenges of communicating through PPE, short amounts of time with individual patients, and language and cultural barriers. Like all care settings, each patient is unique. Among cases presenting for care, the signs, symptoms and in the field even the history of an EVD patient may be non-specific. Suspect case definitions are necessarily sensitive. Good individual and collateral history taking, and the use of systematic testing and empiric treatment protocols addressing common health risks and care challenges are important.

Symptom-control strategies should be adopted early and throughout illness—myalgias, arthralgias, sore throat, abdominal and atypical chest pain, nausea and vomiting, and anxiety are common features that can be addressed in the care of moderately and severely ill EVD patients.

## Engage patients for help when appropriate

Even the best staffed EVD care centers may be challenged in delivering continual care to patients. Tasking patients to drink specific quantities of ORS and reviewing their performance frequently builds rapport, empowers patients, and improves intake volume. When resources become stretched, and sometimes in the best of circumstances, recovering patients may be invaluable as informal aides in the care of others. The EVD care center becomes a microcosm for community organization. Other patients often contribute to the care of pregnant women, young children, and the elderly—encourage this sense of community. It gives patients more control of a daunting care environment. Telephones in the isolation area for patient use can help care and morale. Recovering patients sometimes can be tasked to help monitor the sickest of patients, prepare and coach taking of ORS solution, potentially change IV bags, call health staff, and translate. In other resource-constrained settings, family members have alerted clinicians when IV fluid bags are empty during the resuscitation of a patient.^6^ After recovery, survivors and their families can be invaluable in building community relationships outside treatment centers.

## Beware of the challenges of patient care on the *suspect* ward

Patients present for triage and screening for EVD when they are sick. These patients might have EVD, another severe illness, or both. Admitting a patient into isolation, particularly one not yet confirmed with EVD, provokes a careful risk-benefit analysis for this reason. The suspect ward admits patients awaiting laboratory confirmation of their EVD, or exclusion of EVD as the cause of their illness. Patients may need to stay in the suspect ward for 3 or more days while waiting for reliable diagnostic test results. The objective is to provide sufficient benefit to both the patient and the community to outweigh the risk to the patient if negative. The level of care necessary here can be high. We have observed the manifestations of severe malaria, gastrointestinal bleeding in human immunodeficiency virus (HIV), viral hepatitis co-infected patients with cirrhosis, pulmonary hemorrhage in the setting of severe heart failure, epistaxis caused by malignant hypertension, complicated pregnancy, and non-Ebola viral hemorrhagic fevers.

Use the same caution as in the high-risk area with confirmed EVD patients. However, some—if not many—of the patients admitted to the suspect ward will not be EVD infected and will be discharged rather than admitted to the confirmed ward. Regardless of their EVD infection status, they need a combination of individual patient and epidemiology-directed empiric therapy. Careful practices for patient toileting, patient placement, and triage for communicability and clinician hygiene in between assessing individual suspect patients are important to mitigate patient risk of acquiring EVD infection if not yet infected.^7^

Assessment and management of infants and children pose specific challenges in the field. Occasionally children without symptoms have entered facilities with their ill caregiver, such as the breastfeeding infant of an ill mother. These infants and children, who cannot communicate symptoms clearly yet sometimes move freely around the care center, may need to be monitored with particular attention throughout their time in the care center and for 21 days after release. They will have care, nutrition, and EVD screening needs in and out of isolation. Many of them will fall ill with EVD. When infants and children have been ill with EVD, sometimes a healthy adult has elected to enter to care for them. Healthy adults require considerations similar to those for healthy children.

## Decrease barriers between the patient and the community when appropriate

Isolating a patient introduces a high burden of care and may create social barriers between the outbreak response and patients, families, and their communities. Healthcare providers are wrapped in PPE, giving patients very limited ability to make eye contact or read facial expressions. Patients, their families and communities frequently witness deaths, followed by decontamination of corpses and placement in body bags. These experiences are traumatic and promote not only alienation of the outbreak response from the community, but also increase patients' sense of isolation from caregivers and the outside world.

Ensure that patients have ways to safely communicate with healthcare workers, family, and friends. This can be done using open line of sight areas with low barriers where ambulatory patients can speak with visitors across a safe distance. In well-appointed hospitals, glass and electronic communication devices can be used. Seek mechanisms for patients to charge their cellular phones to allow continued communication with family and friends. Provide positive feedback to families and friends that come to the visitation area. When resources allow and when appropriate and acceptable to the patients, consider family entry into the high-risk area in PPE, escorted for a supervised visit after prior training and indoctrination. Some patients, though, want time before interacting with others. When discussing burial with families of deceased patients, allow for their viewing of the body and participation in a safe burial. This basic respect for patients and families helps to build and maintain positive relationships with communities, overcoming common misunderstandings and making activities in the EVD care center more transparent.

## Balance staff and patient needs; care for each other

Physical and emotional fatigue may contribute to errors in clinical decision making and IPC practice. Staff must protect and monitor their health and the health of their colleagues. Be wary of physical symptoms of dehydration, fatigue, and psychological stresses caused by working in resource-constrained and high-risk environments. This is true at the care site, after hours and after the period providing care. For international staff, the post-deployment period may present additional but under-appreciated stressors. Returning to a higher resourced health care setting leads to an *inequity-tension* experienced by many people working both in low- and high-income countries. Colleagues, neighbors, and others at home may have considerable apprehension about interactions with returning healthcare workers, even though they may have little reason to suspect EVD or other illness. Recently announced quarantine of asymptomatic health care workers in some jurisdictions inevitably adds to this post-deployment stress. We have used our network of consultants not only as a technical sounding board but also for personal support in and away from the field.

Following both general and specific principles, we can provide effective and safe care regardless of geography. In an outbreak, clinicians must focus on the part they play in practicing the best EVD care possible. They also should appreciate that direct patient care is both inextricably linked with the overall outbreak response and only one part of what is necessary to control an outbreak. An effective public health response brings patients to care. Direct clinical care builds trust, which facilitates other elements of the response. Strong surveillance, contract tracing and monitoring, social mobilization, and risk communication are essential. A well-functioning EVD care center promotes the integration of all of these aspects while respecting the broad range of work being accomplished by others.

Clinicians and all staff participating in outbreak response should strive to leave a legacy in improved systems for local outbreak response. Ideally, these will enable effective local responses, obviating the need for deployment rotations for future outbreaks. Until then, *more* prepared, fully equipped, and supported clinical teams are needed to confront EVD in West Africa. Clinical *preparedness* is needed in at risk countries.
